# Exposure to Quaternary Ammonium Compounds (QACs) in Assisted Living Facilities: Implications for Older Adults

**DOI:** 10.1021/acs.est.5c05821

**Published:** 2026-02-03

**Authors:** Minghao Kong, Tret Burdette, Raghu Sanath Kumar, Claire Dempsey, Parinya Panuwet, Amina Salamova

**Affiliations:** † Gangarosa Department of Environmental Health, Rollins School of Public Health, 25798 Emory University, Atlanta, Georgia 30322, United States; ‡ Center for Applied Isotope Studies, 1355 University of Georgia, Athens, Georgia 30602, United States; § Department of Public Health, 2837 Agnes Scott College, Atlanta, Georgia 30030, United States

**Keywords:** quaternary ammonium compounds, indoor exposure, dust, wristbands, indoor air, older adults

## Abstract

Quaternary ammonium compounds (QACs) are commonly used in disinfecting and personal care products for their antimicrobial, surfactant, and preservative properties. This study provides the first comprehensive assessment of QACs in assisted living facilities through the analysis of 19 QACs from three different QAC subgroups in indoor dust and air samples collected from three assisted living facilities in Indiana, United States (US), as well as in wristbands worn by the residents and staff of these facilities. The medians of the total QAC concentrations (∑QAC, the sum of 19 QAC concentrations) were 151,000 ng/g in dust, 3.17 ng/m^3^ in air, and 2,290 ng/g in wristbands. Benzylalkyldimethylammonium compounds (BACs) were the most abundant QAC group in all three matrices and contributed 58–87% to the ∑QAC concentrations. The QAC distribution patterns found in dust, air, and wristbands were similar to those reported for disinfecting products, suggesting these products could be an important indoor source in assisted living. QAC concentrations in wristbands worn by staff during their work shift were significantly higher than those in wristbands worn by residents (*p* < 0.05). In addition, the levels found in dust from assisted living were several times higher than those previously reported in US residential households. Concentrations of C12-, C14-, and C16-BACs in dust, air, and wristbands significantly and positively correlated, suggesting common sources in the indoor environment. Estimated daily intake (EDI) of QACs suggests that accidental dust ingestion is the predominant exposure route, accounting for approximately 62% of the total QAC intake. The elevated QAC concentrations in assisted living facilities are of concern for the residents and staff of these facilities because of the potential health risks associated with exposure to these chemicals, such as respiratory effects.

## Graphical abstract

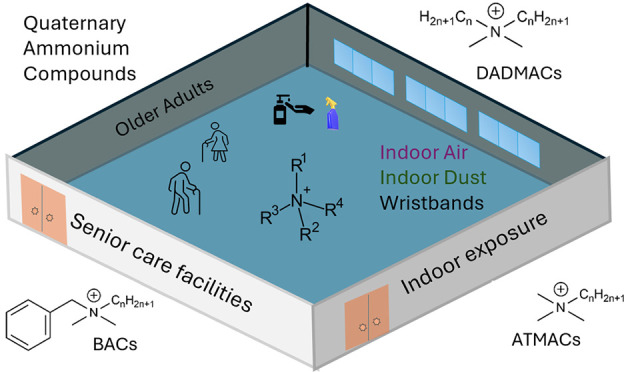

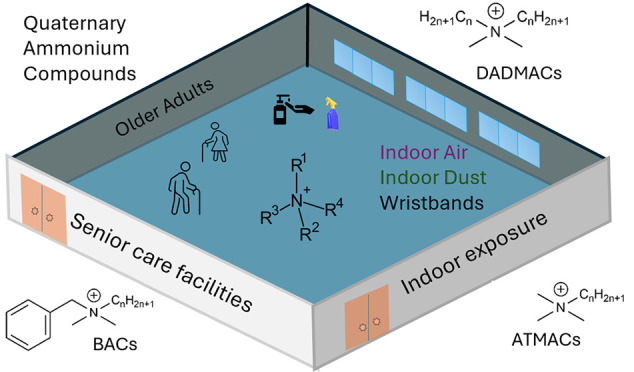

## 1 Introduction

Quaternary ammonium compounds (QACs) are a class of chemicals commonly used in personal care and disinfecting products and textiles for their antimicrobial, surfactant, and preservative properties.^1^ Three QAC subclasses commonly used in disinfecting and personal care products include benzylalkyldimethylammonium compounds (BACs), alkyltrimethylammonium compounds (ATMACs), and dialkyldimethylammonium compounds (DADMACs). BACs, particularly the C12-, C14-, and C16-BACs, are active ingredients in about 670 antimicrobial products registered with the US EPA.^1^ Moreover, QACs are the main ingredients in most of the disinfecting products recommended by the United States (US) Environmental Protection Agency (EPA) for use in residential and public spaces against the SARS-CoV-2 virus, which resulted in an increased use of QACs following the COVID-19 pandemic outbreak.^2–4^ As a result, QAC-containing products are extensively used in various indoor environments, with commercial products often formulated at higher concentrations. Exposure risks may vary with application method: spraying and fogging of products may elevate inhalation exposure, while handling concentrated disinfectant solutions may increase dermal uptake.^1,5^ Postapplication, QACs can persist on surfaces and partition to the indoor air and dust, posing long-term exposure.^6^ This is especially pertinent in healthcare and long-term care facilities, where disinfection is intensive and frequent.

There is a growing body of *in vitro*, *in vivo*, and *ex vivo* studies showing that some QACs can exert a range of toxic effects, including immunotoxicity,^7^ infertility,^8,9^ and developmental toxicity.^9–11^ Human health studies have shown that hospital staff are at increased risk of developing work-related asthma and other respiratory illnesses when exposed to BACs from occupational product use.^12–14^ An eight-year study found that cases of work-related asthma associated with exposure to BACs increased significantly over the observation period.^13^ Recent studies show that the high percentage of the general population have detectable concentrations of several BACs and DADMACs in blood, with significantly higher levels detected after the COVID-19 pandemic.^15–17^ QACs and their metabolites were also recently found in human urine and feces.^18^ This widespread chronic exposure to QACs adds increased concerns of adverse health effects on the general population.

Environmental exposures are especially concerning for the vulnerable population subgroups, such as children and older adults, as these populations are more sensitive to the adverse effects of these exposures due to their physiological and developmental characteristics.^19,20^ We have recently shown that exposure to QACs in nonresidential spaces, such as childcare facilities, is several times higher compared to homes, possibly due to the high use of QAC-containing commercial disinfectants commonly used at daycares and schools.^21–23^ However, there is limited information on QAC exposure affecting older adults, especially those living in senior care facilities. Senior care facilities heavily use surface and spray disinfectants to fight the spread of pathogens.^24,25^ Disinfectant use in these settings has potentially increased during the COVID-19 pandemic in an attempt to prevent the virus from spreading, since an estimated 21% of all COVID-19 deaths in the US were from senior care facilities.^26^ The high usage of QACs and their association with adverse respiratory effects are particularly concerning for seniors since they contract respiratory illnesses more often than younger adults^27^ and increased frailty is linked with poor recovery from the illnesses.^28^ In our previous study with 222 participants, 97% of subjects had detectable concentrations of at least one QAC, and 52% of those were over the age of 60.^15^ This is a concern for older adults due to their weakened immune system and reduced metabolic efficiency.^19,29^ In addition, the significant amount of time they spend indoors typically results in increased exposure to indoor pollutants.^30–32^

In this study, we quantified exposure to 19 QACs in assisted living facilities in Indiana, US. We collected paired samples of indoor dust and air from the residences of participating seniors living in the facilities. We also collected wristbands from the same senior participants, as well as select staff members, to assess personal exposure to QACs. This is the first study assessing environmental and personal exposures to QACs among the residents and staff of assisted living facilities.

## 2 Methods

### 2.1 Sample Collection

Sampling was performed at three assisted living facilities in Indiana, US, between October 2021 and August 2022. A total of 43 residents of these facilities were recruited to participate in this study. Participants were all at least 65 years old at the time of recruitment and had lived at the facility for at least 6 months before participating in this study. The study was approved by the Indiana University Institutional Review Board (Protocol #1909013530) and the participants provided informed consent prior to participating in the study.

Paired samples of indoor dust and air (*n* = 39 pairs) were collected from each participant’s room. Air was sampled using precleaned polyurethane foam (PUF) disks with a diameter of 5 1/2 in. (Tisch Environmental, US) covered with a steel dome using a previously developed sampling method.^33^ PUF air samplers were employed in each room for a 28-day period to collect air with an assumed airflow of 2.9 m^3^/day.^34^ Floor dust was collected from each participant’s room using precleaned nylon bags inserted into the crevice tool of a dedicated vacuum cleaner during vacuuming.^32^ Silicone bands (1.2 cm × 16 cm; surface area: 19.2 cm^2^) were precleaned following a previously developed method^35^ and were worn by each resident participant for 7 days (*n* = 39). Dust and wristband samples were collected at the end of the 28-day PUF deployment period. In addition to residents, some assisted living staff members wore wristbands for 2–6 days during their work shift only (*n* = 12). When not being worn, wristbands were stored at room temperature wrapped in aluminum foil and sealed in zip-lock bags at the workplace. Staff sampling periods were scheduled based on staff availability and were distributed throughout the 28-day resident sampling period. Upon collection, all samples were individually wrapped in aluminum foil, sealed in separate zip lock bags, and stored at −20 °C until analysis.

### 2.2 Sample Extraction

Dust samples were allowed to thaw at room temperature and then homogenized by mixing and passing each sample through a 500 μm mesh size sieve. Afterward, 100 mg of dust was transferred to a 15 mL glass centrifuge tube (VWR, US), spiked with the internal isotopic standard solution, and extracted with 4 mL of acetonitrile through sonication for 30 min. The samples were then centrifuged at 1900 relative centrifugal force (rcf) for 5 min and the supernatant was transferred to a new tube. The residues were then re-extracted using the same method two more times. The combined supernatants were concentrated to near dryness under nitrogen using a TurboVap (Biotage, Sweden), reconstituted with 1 mL acetonitrile, and transferred to autosampler vials (Agilent, US) for instrumental analysis.

PUF disks were first thawed and cut into quarters. One PUF disk quarter was then weighed, transferred to a 50 mL centrifuge tube (Corning, US), and spiked with internal standards. The sample was then sonicated for 30 min fully submerged in 20 mL of acetonitrile, and the supernatant was transferred to a centrifuge tube. The PUF was re-extracted twice more, and all supernatants were combined. After the final extraction, the PUF was pressed against the 50 mL tube walls to squeeze any remaining solvent out. The extract was then concentrated to near dryness under nitrogen using a TurboVap and reconstituted with 1 mL acetonitrile. The resulting extract was filtered using 0.2 μm nylon filters and then transferred to autosampler vials for analysis.

The wristbands were thawed and approximately one-third was cut off to be used for the analysis. The wristband piece was then weighed and cut into approximately 2–4 mm pieces and placed in a 15 mL centrifuge tube. The wristbands were then extracted using the same techniques used for the dust samples.

Information on the chemicals and reagents used can be found in the Supporting Information.

### 2.3 Sample Analysis

The QAC analytes for this study were C6–C18 BACs, C8–C18 DADMACs, and C8–C18 ATMACs. Samples were analyzed using a high-performance liquid chromatograph (HPLC, Agilent 1260 Infinity, Agilent, US) coupled with a triple quadrupole mass spectrometer (LC-MS/MS, Agilent 6460, Agilent, US) operated in the electrospray ionization mode. The separation of the analytes was performed using a XBridge Premier BEH C18 VanGuard FIT column (130 Å 2.5 μm, 4.6 × 100 mm, Waters, US) with a XBridge Premier BEH C18 VanGuard FIT guard column (130 Å 2.5 μm, 3.9 × 5 mm, Waters, US). The instrumental method is described in the Supporting Information. Data acquisition was performed using optimized parameters for each of the analytes (Table S1). Analyte quantitation was performed on MassHunter software (Agilent, US) using an isotope dilution method.

### 2.4 Quality Control and Quality Assurance

Quality control measures were performed throughout the analysis, including procedural and field blanks (Table S2) and spiked samples (Table S3). Method detection limits (MDLs) were calculated as the sum of the average blank levels and three times the blank standard deviation for each analyte (Table S2). Overall, average spike recoveries for 19 QACs in spiked samples ranged from 85 ± 3% to 116 ± 5% for dust, 71 ± 8% to 116 ± 1% for air, and from 72 ± 16% to 122 ± 11% for wristbands (Table S3). All measurements were within the calibration ranges used for each matrix type.

### 2.5 Data Analysis

The statistical analyses were performed using R version 4.4.0. All data were blank corrected by subtracting the average blank concentration for each analyte from the sample concentration of that analyte. Concentrations below the MDL were replaced with the MDL/ $\sqrt{2}$ ^36^ for the downstream statistical analysis. The percent contribution was calculated as the ratio of the median concentration of each QAC to the total QAC concentration (∑QAC, the sum of all 19 analyzed QAC concentrations).

Spearman correlation analysis was used to examine relationships among concentrations in different matrices. Spearman correlations were performed using logarithmically transformed concentrations of analytes detected in at least 50% of the samples for each matrix. QAC levels (normalized per the number of days worn) in wristbands worn by residents were compared with those worn by staff using the Mann–Whitney U test.

Estimated daily intake (EDI) values were calculated to evaluate intake from accidental dust ingestion, inhalation of indoor air, and dermal uptake. eq 1 was used to calculate the EDI.^23,37,38^

1 $$\mathrm{EDI}=\frac{\left(C_{\mathrm{dust}}\times I_{\mathrm{rate dust}}\times F_{\mathrm{uptake}}+C_{\mathrm{dust}}\times \mathrm{BSA}\times \mathrm{DAS}\times F_{\mathrm{skin}}+C_{\mathrm{air}}\times I_{\mathrm{rate air}}\right)\times T}{\mathrm{bw}}$$

where *C* is the median concentration of a QAC in dust (ng/g) or air (ng/m^3^). *I*_rate_ is the QAC intake rate and values of 0.02 g/day and 13.1 m^3^/day were used for the ingestion of dust and inhalation of air, respectively, for adults over 60 years old.^39^ The exposure time per day, *T*, was 0.95 since seniors spend 95% of their day indoors.^30^ Body weight, *bw*, was 75.7 kg, the average of reported weights for adults age 60 and older.^39^ *F*_uptake_ is the uptake fraction of QAC through dust ingestion estimated as 0.8 (unitless), *BSA* is the exposed total body surface area estimated as 1945 cm^2^; *DAS* is the amount of dust adhered to skin estimated as 0.01 mg/cm^2^; and *F*_skin_ is the fraction of QAC absorbed by the skin estimated as 0.48 (unitless).^36^ EDIs were calculated for two exposure scenarios: the average exposure scenario using the median QAC concentrations and the high exposure scenario using the 95th percentile QAC concentrations.

## 3 Results and Discussion

### 3.1 QAC Concentrations

Table 1 shows the detection frequencies (DF), minimum, median, and maximum concentrations for the analyzed QACs in dust, air, and wristbands, and the contribution of each QAC to the total QAC concentration (ΣQAC, the sum of all 19 QACs), and Figure 1 shows the contribution of each QAC to the ΣQAC concentrations for each matrix.

**Figure 1:**
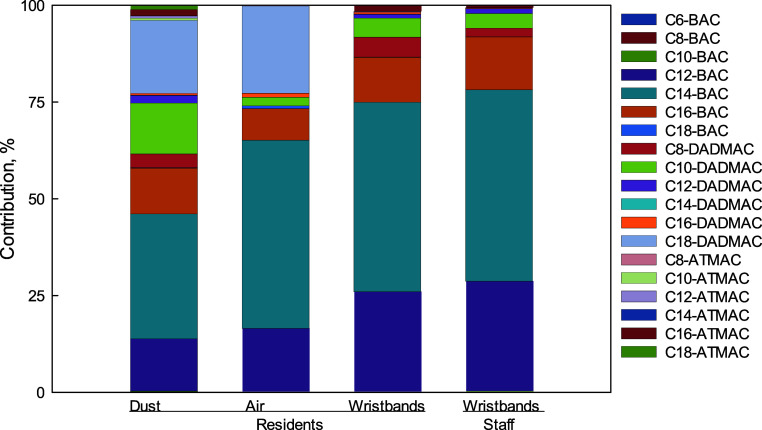
Percent contribution of each individual QAC to the ∑QAC concentration for dust, air, and wristbands (calculated based on median concentrations).

**Table 1:** Summary of the Descriptive Statistics for QAC concentrations in Dust, Air, and Wristband Samples Collected from Indiana Assisted Living Facilities and Their Residents, and Wristbands Collected from Staff^a^

	Residents Dust (*n* = 39)					Residents Air (*n* = 43)					Residents Wristbands (*n* = 39)					Staff Wristbands (*n* = 12)				
	DF	Med	Min	Max	Contr.	DF	Med	Min	Max	Contr.	DF	Med	Min	Max	Contr.	DF	Med	Min	Max	Contr.
	%	ng/g	ng/g	ng/g	%	%	ng/m^3^	ng/m^3^	ng/m^3^	%	%	ng/g	ng/g	ng/g	%	%	ng/g	ng/g	ng/g	%
BACs																				
C6-BAC	85	1.86	<MDL	37.6	0.002	0	-	<MDL	<MDL	-	5	-	<MDL	0.442	-	17	-	<MDL	0.0862	-
C8-BAC	100	138	4.30	1680	0.125	7	-	<MDL	0.0182	-	72	0.423	<MDL	41.7	0.0327	83	1.04	<MDL	40.8	0.0637
C10-BAC	100	181	9.22	4360	0.163	21	-	<MDL	0.00935	-	95	1.24	<MDL	438	0.0961	100	3.30	0.254	131	0.201
C12-BAC	100	15000	2110	90200	13.6	93	0.336	<MDL	2.87	16.5	100	334	27.2	3290	25.9	100	466	55.9	2030	28.4
C14-BAC	100	35700	5570	157000	32.3	95	0.990	<MDL	10.5	48.7	100	632	54.1	7580	49.0	100	811	346	4660	49.5
C16-BAC	100	13000	1390	65500	11.8	91	0.166	<MDL	3.74	8.16	97	150	<MDL	1750	11.6	100	223	99.2	1140	13.6
C18-BAC	97	161	<MDL	2800	0.146	53	0.0150	<MDL	0.388	0.738	90	1.18	<MDL	181	0.0917	100	0.949	0.412	22.6	0.0579
∑BAC		61200	9100	308000	58.1		1.65	<MDL	15.9	74.1		1280	92.1	13100	86.7		1590	559	8000	91.9
DADMACS																				
C8-DADMAC	100	3940	293	26300	3.56	14	-	<MDL	0.144	0.0	82	66.3	<MDL	3090	5.14	92	36.3	<MDL	204	2.22
C10-DADMAC	100	14500	744	80200	13.1	93	0.0444	<MDL	2.76	2.18	97	63.0	<MDL	1390	4.88	100	62.2	7.75	2370	3.79
C12-DADMAC	100	2190	249	18700	1.98	19	-	<MDL	13.2	0.0	97	12.4	<MDL	167	0.961	100	20.1	5.05	206	1.23
C14-DADMAC	92	121	<MDL	1130	0.109	44	-	<MDL	0.0260	-	92	1.62	<MDL	31.6	0.126	83	0.529	<MDL	2.32	0.323
C16-DADMAC	79	462	<MDL	28000	0.418	81	0.0219	<MDL	0.286	1.07	87	5.64	<MDL	513	0.437	83	2.85	<MDL	51.7	0.174
C18-DADMAC	85	20900	<MDL	302000	18.9	86	0.458	<MDL	26.7	22.5	38	-	<MDL	31000	-	17	-	<MDL	6620	-
∑DADMAC		50700	3250	317000	38.1		0.655	<MDL	27.3	25.8		524	151	31800	11.5		280	185	7640	7.45
ATMACs																				
C8-ATMAC	95	39.2	<MDL	453	0.035	79	0.00395	<MDL	0.0161	0.194	23	-	<MDL	5.71	-	33	-	<MDL	1.24	-
C10-ATMAC	100	641	67.3	10700	0.580	2	-	<MDL	0.0574	-	8	-	<MDL	20.2	-	17	-	<MDL	26.7	-
C12-ATMAC	100	603	136	30700	0.545	23	-	<MDL	4.88	-	85	2.36	<MDL	45.2	0.183	58	1.07	<MDL	35.9	0.0653
C14-ATMAC	100	90.4	9.98	805	0.0818	30	-	<MDL	0.273	-	64	0.837	<MDL	19.5	0.0648	67	0.623	<MDL	6.92	0.0380
C16-ATMAC	100	1780	214	9820	1.61	40	-	<MDL	1.21	-	79	20.0	<MDL	780	1.55	100	9.40	3.84	152	0.573
C18-ATMAC	97	1130	<MDL	7650	1.02	5	-	<MDL	0.0455	-	41	-	<MDL	289	-	25	-	<MDL	96.9	-
∑ATMAC		5560	849	40400	3.87		0.282	<MDL	5.10	0.194		38.5	11.7	816	1.80		19.3	12.3	188	0.677
∑QAC		151000	17900	478000			3.17	<MDL	28.1			2290	264	36400			1880	786	11500	

a Detection frequencies (DF, %), minimum (min), median (med), and maximum (Max) concentrations, and the contribution of each individual QAC to the total QAC concentration (Contr., %) are included.

#### 3.1.1 Dust

All targeted QACs were detected in dust samples from Indiana assisted living facilities, with 16 out of 19 QACs detected in more than 90% of the samples (Table 1). The three less frequently detected QACs were C6-BAC and C16- and C18-DADMACs (DF 79–85%). The ΣQAC concentrations in dust ranged from 17,900 to 478,000 ng/g with a median concentration of 151,000 ng/g. The most abundant QAC groups in these samples were the BACs and DADMACs, detected at median concentrations of 61,200 ng/g for the total BAC concentrations (∑BAC) and 50,700 ng/g for the total DADMAC concentrations (∑DADMAC), contributing 58.1% and 38.1% to the ∑QAC concentrations, respectively. ATMACs were found at much lower levels (median total ATMAC concentration [∑ATMAC] 5,560 ng/g) and contributed only 3.87% to the ∑QAC concentration. The dominance of BACs and DADMACs aligns with the QAC patterns observed in indoor dust studies from US and Europe that reported contributions of BACs and DADMACs to the ΣQAC concentrations as 56% and 26%^2^ and 46% and 27%,^40^ respectively. These similar distribution patterns of QACs from different studies suggest similar indoor sources of QACs, such as the applications of disinfectant wipes and sprays containing QACs. Among BACs, C12-, C14-, and C16-BACs were the most abundant and all together contributed 57.7% to the ΣQAC concentrations. These three BACs are the most common ingredients in disinfecting products which may explain their abundance in the indoor environment. The US EPA lists the majority of products registered as antimicrobials as containing C12-, C14-, and C16-BACs as the main ingredients (Figure S1).^1,41^ In contrast, among DADMACs, the longer chain C18-DADMAC was the major DADMAC detected in these samples and constituted 18.9% of the ΣQAC levels. Longer chain DADMACs are generally used in disinfecting products and in personal care products, including hair conditioners.^1,42–44^

The levels of QACs in dust in this study were 3 to 4 times higher than those previously reported in residential house dust collected from homes in Indiana before and after the COVID-19 pandemic (medians 36,300 ng/g and 58,900 ng/g, respectively; Figure 2).^2^ A different cohort of Indiana homes were also tested during the pandemic and measured similar concentrations to those in the first study, with a median concentration of 56,900 ng/g in dust.^17^ Furthermore, these levels are up to 10 times higher than those reported for samples collected from homes and public spaces in Europe (median 14,700 ng/g)^40^ and China (median 42,200 ng/g) in 2022.^45^ ATMAC concentrations in senior living facilities were similar to those in residential dust (6.4–8.8 μg/g), but the overall contribution of ATMACs to the ΣQAC concentration was lower in senior facilities, compared to homes (18–27.5%).^2^ However, the levels found in dust from assisted living facilities were similar to those we recently reported for daycares in the US (median ∑QAC for the same 19 QACs 150,000 ng/g)^23^ and are likely attributable to the extensive application of disinfectants and antimicrobial agents for sanitation purposes in nonresidential public settings serving vulnerable populations, such as childcare and senior care facilities.^1,24,25^ Similarly, the Burdette et al. (2024) study reported nearly twice the concentration of perfluorooctanesulfonic acid (PFOS) in dust from senior care facilities (13 ng/g) compared to residential dust (5.9 ng/g).^31^ A separate pilot study found elevated levels of other environmental contaminants in dust from senior care facilities, including organophosphate esters (mean 24,200 ng/g), polycyclic aromatic hydrocarbons (PAHs) (mean 37,600 ng/g), and brominated flame retardants (3,830 ng/g).^32^ It is noteworthy to mention that the levels of QACs in dust were several orders of magnitude higher than the levels of these other previously reported contaminants. These findings highlight the importance of investigating QAC exposure in senior care facilities and its impact on the health of older adults.

**Figure 2:**
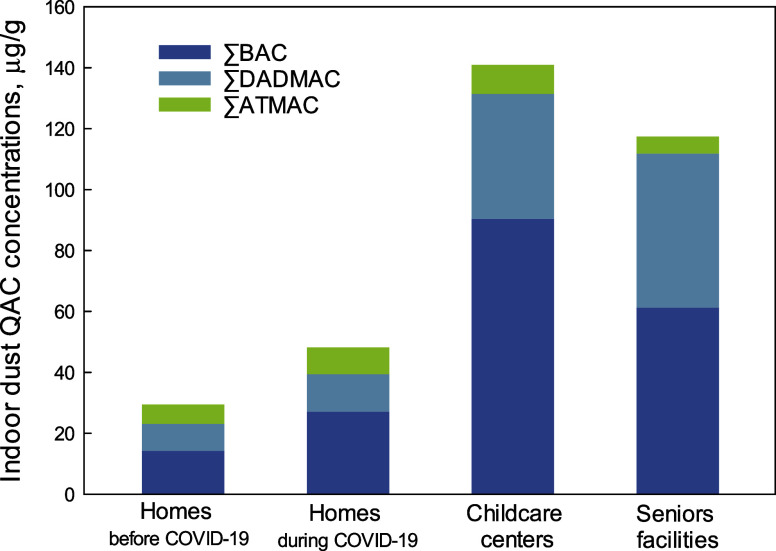
Median ΣQAC concentrations in dust collected from Indiana homes (before and during the COVID-19 pandemic),^2^ childcare centers,^23^ and from assisted living facilities from this study.

#### 3.1.2 Indoor Air

Among the 19 QACs analyzed, 18 were detected in indoor air samples with detection frequencies ranging from 2 to 95%. The ΣQAC concentrations in air ranged from < MDL to 28.1 ng/m^3^, with a median of 3.17 ng/m^3^. Similar to dust, BACs were the most abundant QAC group in air (median ∑BAC 1.65 ng/m^3^) and accounted for 74.1% of the ΣQAC concentrations. Specifically, the most abundant BACs were C12-BAC (16.5% contribution) and C14-BAC (48.7% contribution). DADMACs were the second most abundant group (median ∑DADMAC 0.655 ng/m^3^) and contributed 25.8% of the ΣQAC concentrations in air with the C18-DADMAC accounting for 22.5%. This pattern was similar to that in dust where C18-DADMAC constituted 18.9% of the ∑QAC dust concentrations. The overall presence of ATMACs in air samples was minimal. The most frequently detected ATMAC was C8-ATMAC (79% DF), which contributed 0.194% to the ΣQAC concentrations. This finding was different from the previously reported preliminary results from air samples collected in homes in Indiana, US (*n* = 6) that showed ATMACs as the predominant QAC group found in air, constituting 78% of the ΣQAC concentrations reported in that study.^15^ However, the latter study measured a similar median concentration of QACs in air (3.29 ng/m^3^). Different occurrences of ATMACs in senior care and residential homes may be due to higher use of products containing ATMACs in homes, such as air fresheners. When compared to other chemical groups, the concentrations of QACs in air were 60, 5, and 7 times higher than those of polybrominated diphenyl ethers, polychlorinated biphenyls, and organochlorine pesticides, respectively, but lower than those of PAHs reported previously in indoor air.^46^

#### 3.1.3 Wristbands

Among 19 QACs, 8 were detected in 90–100% of wristbands, 4 were detected in 75–90% of wristbands, and the remaining 7 were detected less frequently (Table 1). The ΣQAC concentrations in wristbands ranged from 264 to 36,400 ng/g with a median of 2,290 ng/g. Similar to dust and air, BACs accounted for the majority of the ΣQAC concentrations in wristbands (86.7% contribution to the ∑QAC concentrations), followed by DADMACs (11.5% contribution) and ATMACs (1.80% contribution). C12-, C14-, and C16-BACs were the most abundant BACs found on wristbands and contributed 25.9, 49.0, and 11.6% to the ΣQAC concentrations, respectively. The higher relative abundance of BACs in wristbands (Figure 1) compared to dust and air may be due to differences in partitioning of QACs onto wristbands, and in exposure pathways and sources of the QACs. Wristbands, as passive sampling media, reflect time-weighted and vapor-phase concentrations, capturing volatile/semivolatile organic contaminants and reflecting dermal and inhalation routes.^47–49^ These differences may also stem from the presence of the aryl group in BACs, which tends to adsorb onto silicone wristbands.^47^ In contrast, DADMACs and ATMACs may not be as effectively sampled by silicone, as compared to textile materials like cotton and rayon.^50^ Similarly, relatively low levels and infrequent detections of nonvolatile PFOS and perfluorooctanoic acid (PFOA) in silicone wristbands, compared to volatile per- and polyfluoroalkyl substances (PFAS) were also reported with the chemical uptake rates being a potential reason.^51^ The Burdette et al. study also found that the nonvolatile PFAS were detected in wristbands with low frequency (most <40%) and at low concentrations (maximum concentration of the most detected PFOA, 0.12 ng/g),^31^ while Hoxie et al. reported more frequent detection for more volatile PFAS and at higher concentrations (up to 15.9 ng/g).^51^ Given that silicone wristbands are selective for volatile, semivolatile, and aromatic compounds,^31,51^ we hypothesize that the lower levels of DADMACs and ATMACs compared to BACs are due to the wristbands’ limited sampling capacity. To test this hypothesis and better understand the variations from exposure or the wristband’s compatibility, further comparisons with other types of wristbands, such as those made from high-density polyethylene, thermoplastic polyurethane, cotton, and rayon, are necessary.

The concentrations of QACs normalized per the number of days worn (Table S5) in wristbands worn by the assisted living residents were compared to those of the staff at the facilities. The wristbands from staff members exhibited higher detection frequencies and concentrations for most BAC congeners compared to the seniors (Table 1). Notably, C8–C16 BAC concentrations were significantly higher in staff wristbands (*p* < 0.05) as shown in Figure 3. DADMACs were detected in most wristbands but were found at lower concentrations in staff members overall, with the exception of C12-DADMAC that was found at significantly higher levels in staff wristbands compared to the residents. ATMAC concentrations were generally lower in staff wristbands as well, and no statistically significant differences were found for ATMACs in senior and staff wristbands. Overall, the median ∑BAC concentration in staff wristbands was 24% higher compared to that in seniors’ wristbands, which could be attributed to higher occupational exposure from frequent cleaning and disinfection practices. The elevated levels of BACs raise concerns about higher exposure among staff, including respiratory and other long-term health effects.^12,14,52,53^

**Figure 3:**
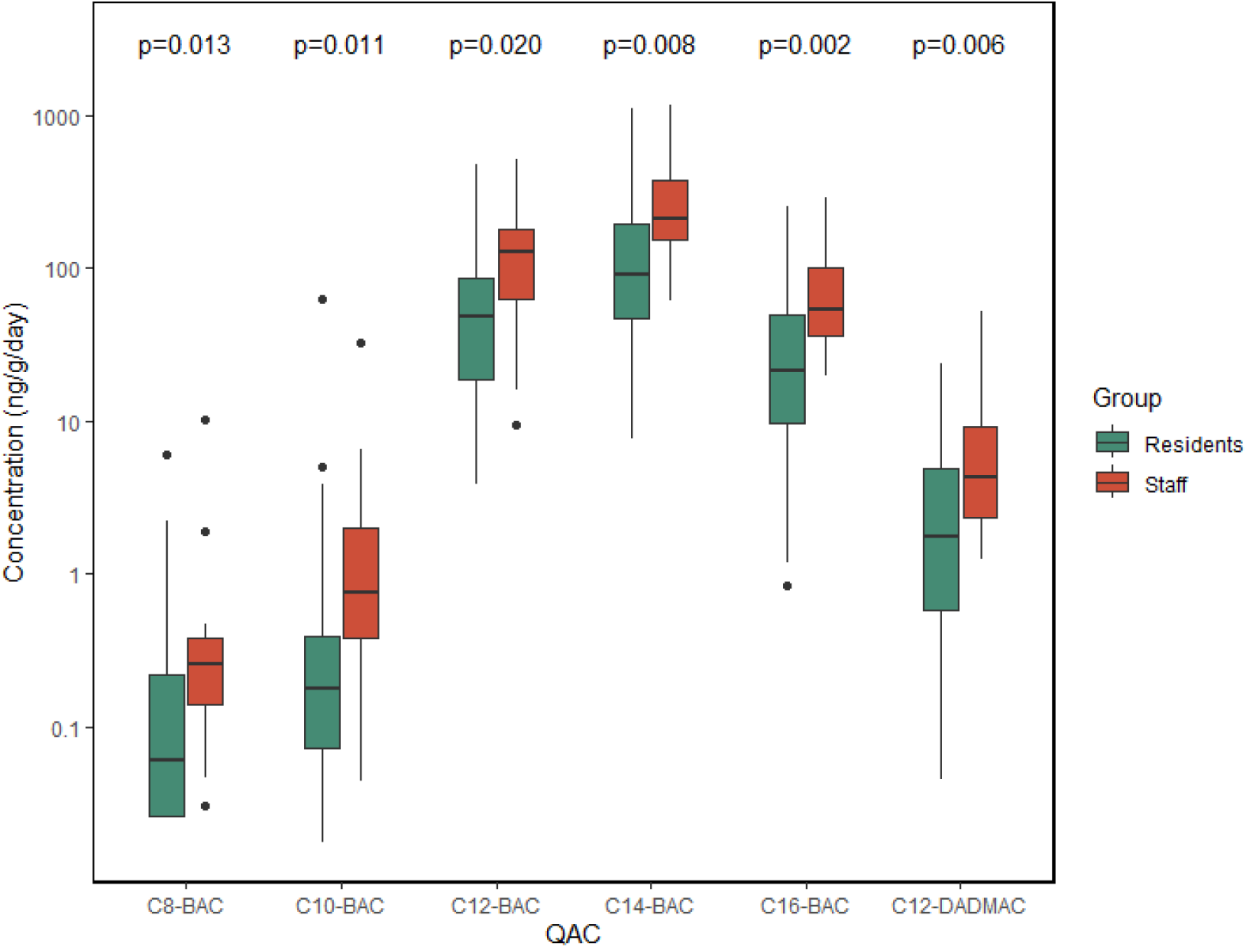
Comparison of the QAC concentrations (ng/g/day) in wristbands from residents and staff of the assisted living facilities (Mann–Whitney U test).

### 3.2 Concentration Correlations

Correlations between the logarithmically transformed concentrations of QACs detected in more than 50% of the samples within and across matrices were examined using Spearman correlations analysis. The results of the analysis are presented in Figure 4 and Table S4.

**Figure 4:**
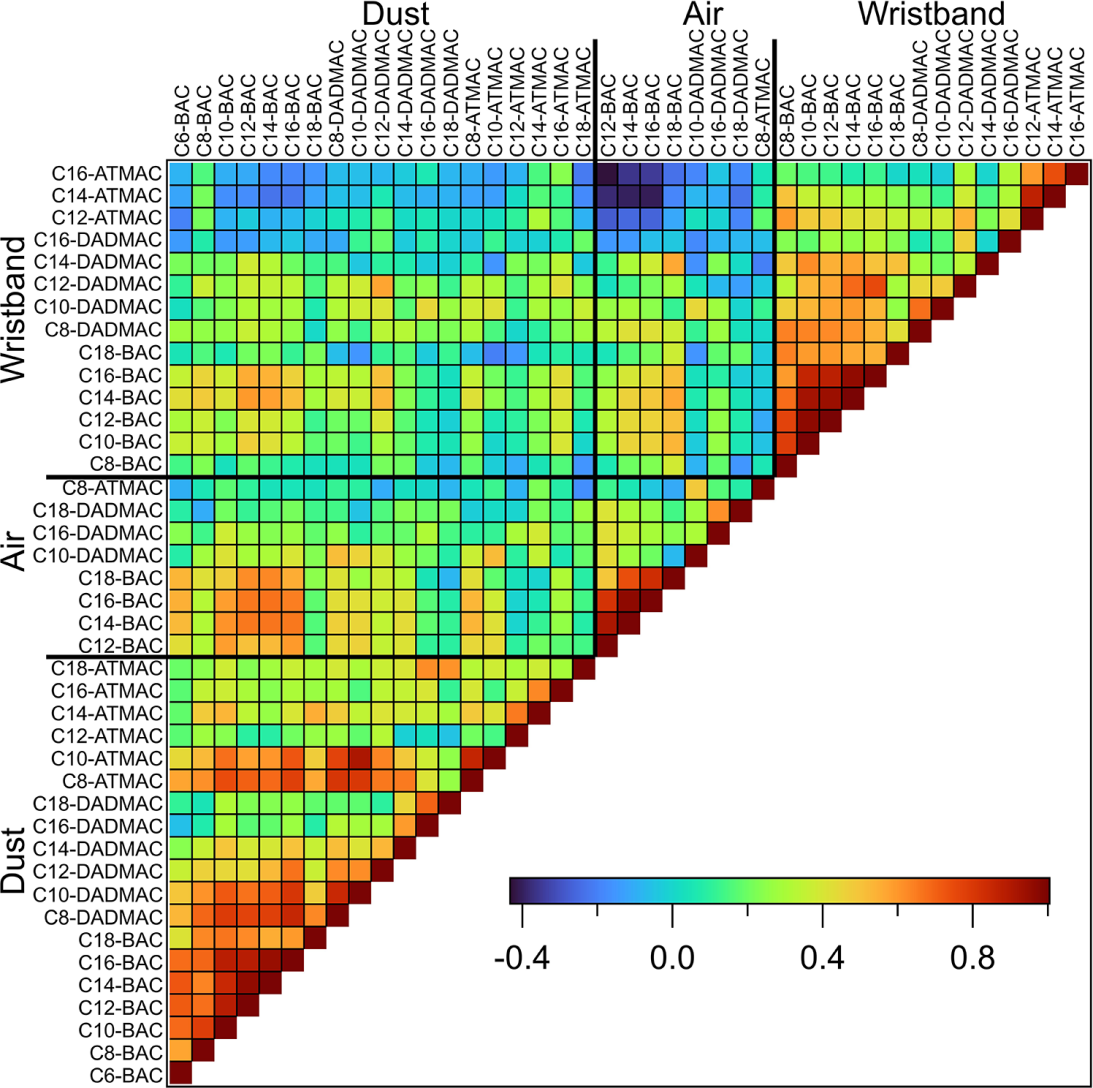
Spearman correlations among QAC concentrations in dust (D), air (A), and wristbands (WB) collected from the residents of assisted living facilities.

The most notable significant positive correlations were observed among C12-, C14-, and C16-BACs, including strong within-matrix correlations in dust (ρ: 0.88–0.96, *p* < 0.01), wristbands (ρ: 0.88–0.97, *p* < 0.01), and indoor air (ρ: 0.78–0.96, *p* < 0.01). Similarly, these three QACs showed significant positive correlations between matrices (ρ: 0.32–0.64, *p* < 0.05) except C12-BAC in dust and C16-BAC in air (ρ: 0.22, *p* = 0.21). In general, all BACs shared at least one significant correlation among matrices. These findings suggest that BACs in these matrices likely originate from similar sources but may undergo different transport and accumulation mechanisms. Interestingly, C18-BAC in dust exhibited no significant correlation with BACs in other matrices, suggesting the possibility of a distinct source or physiochemical properties compared to other BAC congeners. Alternatively, the lack of significant relationships among the concentrations in different matrices may also be due to the differences in sampling periods for dust and air (28 days) and wristbands (7 days).

DADMACs with longer carbon chains (dominated by C18-DADMAC) and shorter carbon chains (dominated by C10-DADMAC) had different correlation patterns. Short-chain DADMACs (C8–C12) exhibited moderate to strong positive correlations within and across matrices, implying similar accumulation patterns and potential common sources. In contrast, longer chain DADMACs, especially C18-DADMAC, displayed limited intermatrix correlations, suggesting different transport mechanisms, distinct emission sources compared to short-chain DADMACs, or variations in physiochemical properties. This was an interesting variation within the DADMAC subclass and may indicate differences in the accumulation and sources of the longer vs shorter-chain DADMACs. Notably, longer-chain DADMACs, such as C18-DADMAC, are commonly used as antimicrobials in personal care products, particularly in hair conditioners, which may influence their environmental distribution.

Unlike BACs and DADMACs, ATMACs exhibited weak or nonsignificant correlation across matrices. Interestingly, C16-ATMAC in wristbands had a weak but statistically significant negative correlation with C12-, C14-, and C16-BACs in air samples (ρ: −0.45 to −0.39, *p* < 0.05). C12-ATMAC in wristbands also had negative correlations with BAC congeners in air, but they were not significant. This suggests that ATMACs and BACs may interact differently in indoor environments, potentially due to their varying volatility, adherence properties, or usage patterns.

Overall, wristbands showed fewer correlations with dust and air. This discrepancy may be influenced by the sampling capacity and intake rate of wristbands, which could vary depending on the chemical properties of the compounds or limited recovery of QACs from the wristbands (Table S3). In particular, the limited sampling capacity of wristbands may explain the weaker correlations observed for ATMACs, as these compounds may have different adherence or absorption characteristics compared to BACs and DADMACs.

### 3.3 Estimated Daily Intake (EDI)

The EDIs of QACs through accidental dust ingestion, indoor air inhalation, and dermal dust absorption were calculated using the median (average exposure scenario) and 95th percentile (high exposure scenario) concentrations of the QACs detected in at least 50% of the dust and air samples and are shown in Table 2. The ∑QAC EDI through dust ingestion was estimated as 30.2 ng/kg bw/day and was determined as the predominant exposure route for older adults. It constituted nearly 62% of the total estimated intake (the sum of dust ingestion, inhalation, and dermal uptake) and was followed by dermal absorption through skin (17.6 ng/kg bw/day, 36.4%). Inhalation contributed minimally (estimated as 0.521 ng/kg bw/day, 1% contribution), likely due to the general low volatility of QACs. For dust ingestion, the ∑QAC EDIs of seniors were comparable to those of adults who engaged in more frequent disinfecting during COVID-19 (52.7 ng/kg bw/day) but were significantly lower than the EDIs of toddlers attending US daycares (206–615 ng/kg bw/day).^2^ QACs have strong adherence to dust and persist on indoor surfaces, supporting our finding of dust ingestion as the dominant exposure route.^54^

**Table 2:** Estimated Daily Intakes (EDI, ng/kg bw/day) of QACs in Older Adults via Dust Ingestion, Air Inhalation, and Dermal Contact Estimated Based on Median (Average Exposure Scenario) and 95th Percentile (High Exposure Scenario) Concentrations^b^

	Average Exposure Scenario				High Exposure Scenario			
	Dust Ingestion	Inhalation	Dermal Absorption	Total EDI^a^	Dust Ingestion	Inhalation	Dermal Absorption	Total EDI^a^
BACs								
C12-BAC	3.012	0.055	1.76	4.82	15.0	0.206	8.73	23.9
C14-BAC	7.17	0.163	4.18	11.5	25.4	0.650	14.8	40.8
C16-BAC	2.62	0.027	1.53	4.17	11.9	0.148	6.93	18.9
C18-BAC	0.032	0.002	0.025	0.050	0.100	0.012	0.059	0.170
∑BAC	12.3	0.270	7.17	19.7	51.0	1.01	29.7	81.7
DADMACS								
C10-DADMAC	2.91	0.007	1.70	4.62	9.82	0.055	5.73	15.6
C16-DADMAC	0.093	0.004	0.054	0.150	1.96	0.017	1.14	3.11
C18-DADMAC	4.19	0.075	2.45	6.71	42.7	0.204	24.9	67.9
∑DADMAC	10.2	0.108	5.94	16.2	53.5	0.549	31.2	85.2
ATMACs								
C8-ATMAC	0.008	0.001	0.005	0.013	0.046	0.001	0.027	0.073
∑ATMAC	1.12	0.046	0.651	1.81	4.88	0.414	2.85	8.14
∑QAC	30.2	0.521	17.6	48.4	81.2	2.90	47.4	132

a For reference, the tolerable daily intake (TDI) established by the European Food Safety Authority for BACs and DADMACs is set at 1 × 10^5^ ng/kg bw/day.^55^

b Sum of dust ingestion, inhalation, and dermal absorption EDIs.

For individual QACs, C14-BAC had the highest total EDI of 11.5 ng/kg bw/day, followed by C18-DADMAC (6.71 ng/kg bw/day). Similar to the pattern observed for ∑QACs, estimates of exposure to individual QACs were also dominated by dust ingestion, with dermal uptake as the secondary pathway. The EDI for the ∑BACs was estimated at 19.7 ng/kg bw/day), followed by the EDI for the ∑DADMACs (16.2 ng/kg bw/day). For ATMACs, intake was substantially lower, with the total ΣATMAC EDI estimated as 1.81 ng/kg bw/day. The overall EDI through all three routes for the ΣQAC concentration was estimated as 48.4 ng/kg bw/day, which was below the 1 × 10^5^ ng/kg bw/day tolerable daily intake (TDI) established by the European Food Safety Authority (EFSA) for BACs and DADMACs, indicating relatively low overall risk.^55^

Overall, dust ingestion and dermal uptake were estimated to be the main routes of QAC exposure, consistent with previous studies.^6,56^ These findings emphasize that contact with treated surfaces, personal care products, and textiles may play a critical role in QAC exposure. While the total intake remains significantly below regulatory limits, the high reliance on disinfectants and personal care products results in chronic low-level exposure, warranting further investigation into potential long-term health effects.

## 4 Strengths and Limitations

This study is the first comprehensive assessment of QAC exposure in senior care facilities. Our results show that exposure to several QACs is significantly higher in senior care facilities compared to residential homes. In addition, the QAC concentrations in dust, wristbands, and air from these assisted living facilities were several orders of magnitude higher compared to other common indoor contaminants. The dominant QAC groups, BACs and DADMACs, accounted for 96% of the ∑QAC concentrations, a pattern consistent with that in disinfectants and personal care products. This indicates that seniors residing in these facilities experience sustained, high-level exposure to QACs, warranting further research into potential long-term health effects. However, this study also had some limitations. The study was performed within a small geographic region on a small cohort of participants. We did not collect information on the use of QAC-containing products by residents or staff. The small sample size may have limited the correlations between the concentrations in different matrices. Analytical methods (i.e., wristband analysis) used in this study should be further validated in future studies. The study also lacked biomonitoring of participants, which could further explain how environmental exposure affects bioaccumulation of QACs. These factors should be considered when interpreting the results of this research. Overall, our results warrant future studies on biomonitoring of QACs with specific focus of measuring QACs and their metabolites in human blood, urine, and feces. Future studies should focus on comprehensive health risk assessments tailored to vulnerable elderly populations, considering the cumulative impact of chronic, low-level QAC exposure in these indoor environments.

## Supplementary Material



## Notes

List of target analytes, additional details of the instrumental methods, and quality control and quality assurance data, including MDLs, blank concentrations, and spiked sample recoveries; results of the Spearman correlation analyses; staff wristband concentrations (ng/g/day) (PDF)
