# Supplementary material for: Exposure to Quaternary Ammonium Compounds (QACs) in Assisted Living Facilities: Implications for Older Adults
Source: Environ Sci Technol. 2026 Feb 3;60(6):4516–25. doi: 10.1021/acs.est.5c05821 (PMC12918522; doi:10.1021/acs.est.5c05821)
Supplement: Supplementary file 1 [file es5c05821_si_001.pdf]

SUPPORTING INFORMATION

Exposure to Quaternary Ammonium Compounds (QACs) in Assisted Living Facilities: Implications for Older Adults

Minghao Kong[1‡], Tret Burdette[2‡], Raghu Sanath Kumar[1], Claire Dempsey[3], Parinya Panuwet[1], Amina Salamova[1*]

[1] Gangarosa Department of Environmental Health, Rollins School of Public Health, Emory University, Atlanta, GA 30322, USA

[2] Center for Applied Isotope Studies, University of Georgia, Athens, Georgia 30602, USA

[3] Department of Public Health, Agnes Scott College, Atlanta, GA 30030

[‡]These authors contributed equally to this work.

Number of Pages: 11

Number of Tables: 5

Number of Figures: 1

Chemicals and Reagents

The QAC analytes for this study consisted of C6-C18 BACs, C8-C18 ATMACs, and C8-C18 DADMACs. Native standards for all QACs were purchased through Sigma-Aldrich (MO, USA). Labeled standards for C12-BAC, C14-BAC, C16-BAC, C18-BAC, C10-ATMAC, C12-ATMAC, C10-DADMAC, C12-DADMAC and C18-DADMAC were used for an internal standard solution (ISTD). The labeled standards were purchased from Toronto Research Chemicals and CDN Isotopes Inc (QC, Canada). The ISTD was spiked into each sample at an amount of 20 ng. High performance liquid chromatography (HPLC) grade methanol (Fisher Chemical, PA, USA), acetonitrile (Sigma-Aldrich), and Milli-Q water (Millipore, Germany) were used for this study. Formic acid was purchased from Thermo Scientific (MA, USA).

Quality Assurance and Quality Control

Quality assurance and quality control (QC/QC) procedures were followed for this study and included field and laboratory blanks and spiked samples for each sample type. Field blanks were collected during the sampling of the wristband and air samples by briefly keeping the pre-cleaned PUF and wristbands open to the air during collection of the wristband and air samples on specific days. In addition, laboratory blanks were included with each batch of samples that were analyzed in the laboratory. Laboratory blanks for dust included spiking a 15 mL centrifuge tube with the ISTD solution; for air samples, ISTD was spiked to a precleaned polyurethane foam (PUF) disk. All blanks were extracted and analyzed following the same protocols as the samples. In addition, spiked samples were added to each batch of samples and consisted of spiking of 5 ng and 50 ng (separately) of a native QAC solution to pooled dust, pre-cleaned wristbands, and PUF

disks. Spiked matrix samples were analyzed following the same procedures as the samples. A consistent calibration range (0.05–200.00 ng) was used for dust, air, and wristbands, based on prior QAC studies and validated to capture expected levels. Sample amounts were optimized to stay within range. Linearity was confirmed per analyte using 10-point curves, accepting only those with $r^2 > 0.995$ to ensure quantification accuracy.

Instrumental Analysis

MRM transitions of native and labeled (L) QACs and other instrumental parameters if the analytical method used in this study are provided in Table 1. The solvents used for the LC gradient were ultrapure water (solvent A) and methanol (solvent B) that both contained 25 mM ammonium formate and 0.3% formic acid. A solvent delay was implemented using a ZORBAX RR Eclipse Plus C18 delay column (95 Å 3.5 µm, 4.6 x 50 mm, Agilent, USA). The gradient used started with an 80:20 ratio of solvent A to solvent B at a flow rate of 0.7 mL/min. The ratio changed to a 40:60 ratio over the first 0.5 min and held at that ratio until 4 min into the run. The ratio then changed to a 25:75 ratio and held at this until 11 min of run time. The gradient then shifted to a 10:90 ratio and maintained this until reaching 16 min and then shifted to a 0:100 ratio. The 100% solvent B flow ran until 21 min into the analysis at the initial 0.7 mL/min flow rate and then shifted to a 1.2 mL/min flow rate which was maintained until the 29 min point of the analysis collect the final analytes and clean the column. Finally, the solvent ratio returned to 80:20 and the flow rate reduced to 0.7 over a 0.5 min period and the ratio and rate were maintained until the 33 min mark to prepare the column for the next sample.

Table S1: Instrument parameters for instrumental analysis using MRM transitions of native and labeled (L) QACs.

Abbreviation	Compound	Labeled standards used for quantification	Fragment or (V)	Precursor ion (m/z)	Quantification		Confirmation	
					Product ion (m/z)	Collision energy (eV)	Product ion (m/z)	Collision energy (eV)
C6-BAC	C6 Benzylalkyldimethyl Ammonium Compound	C12-BAC (L)	109	220	91	24	65	65
C8-BAC	C8 Benzylalkyldimethyl Ammonium Compound	C12-BAC (L)	106	248	91	40	58	28
C10-BAC	C10 Benzylalkyldimethyl Ammonium Compound	C12-BAC (L)	109	276	91	32	58	28
C12-BAC	C12 Benzylalkyldimethyl Ammonium Compound	C12-BAC (L)	139	304	91	32	58	44
C12-BAC (L)		NA	99	311	98	52	-	-
C14-BAC	C14 Benzylalkyldimethyl Ammonium Compound	C14-BAC (L)	99	322	91	40	58	36
C14-BAC (L)		NA	96	339	98	56	-	-
C16-BAC	C16 Benzylalkyldimethyl Ammonium Compound	C16-BAC (L)	99	360	91	48	58	36
C16-BAC (L)		NA	86	367	98	36	-	-
C18-BAC	C18 Benzylalkyldimethyl Ammonium Compound	C18-BAC (L)	109	388	91	40	58	36
C18-BAC (L)		NA	81	395	98	48	-	-
C8-DADMAC	C8 Dialkyldimethyl Ammonium Compound	C12-BAC (L)	205	270	158	24	71	28
C10-DADMAC	C10 Dialkyldimethyl Ammonium Compound	C10-DADMAC (L)	220	326	57	36	186	32
C10-DADMAC (L)		NA	220	332	192	32	-	-
C12-DADMAC	C12 Dialkyldimethyl Ammonium Compound	C12-DADMAC (L)	124	382	57	44	214	36
C12-DADMAC (L)		NA	114	407	66	48	-	-
C14-DADMAC	C14 Dialkyldimethyl Ammonium Compound	C12-DADMAC (L)	114	438.5	242	40	58	48
C16-DADMAC	C16 Dialkyldimethyl Ammonium Compound	C12-DADMAC (L)	114	494.6	57	56	270	44
C18-DADMAC	C18 Dialkyldimethyl Ammonium Compound	C18-DADMAC (L)	96	550.6	57	56	298	48
C18-DADMAC (L)		NA	142	624.6	336.6	62	-	-
C8-ATMAC	C8 Alkyltrimethyl Ammonium Compound	C10-ATMAC (L)	162	172	60	24	60	24
C10-ATMAC	C10 Alkyltrimethyl Ammonium Compound	C10-ATMAC (L)	109	200	60	28	60	28
C10-ATMAC (L)		NA	182	209	69	28	-	-
C12-ATMAC	C12 Alkyltrimethyl Ammonium Compound	C12-ATMAC (L)	114	228	60	28	85	29
C12-ATMAC (L)		NA	86	231	63	28	-	-
C14-ATMAC	C14 Alkyltrimethyl Ammonium Compound	C12-BAC (L)	109	256	60	28	71	24
C16-ATMAC	C16 Alkyltrimethyl Ammonium Compound	C14-BAC (L)	109	284	60	32	71	28
C18-ATMAC	C18 Alkyltrimethyl Ammonium Compound	C12-DADMAC (L)	220	312	60	36	57	36

Table S2: Average concentrations of QAC analytes in blanks (n = 5 for dust; n = 6 for air; n = 6 for wristbands).

Analyte	Dust (ng/g)			Air (ng/m^3)			Wristband (ng/g)		
	Average conc.	Std dev.	MDL*	Average conc.	Std dev.	MDL	Average conc.	Std dev.	MDL
C6-BAC	0.06	0.12	0.50	ND	-	0.01	ND	-	0.04
C8-BAC	0.12	0.17	0.64	ND	-	0.01	0.03	0.08	0.26
C10-BAC	ND	-	0.50	ND	-	0.01	0.02	0.05	0.18
C12-BAC	3.43	2.61	11.3	0.06	0.02	0.12	3.93	3.64	14.9
C14-BAC	6.04	2.24	12.8	0.12	0.05	0.27	10.4	10.5	41.9
C16-BAC	2.40	1.39	6.59	0.03	0.01	0.06	2.28	2.05	8.44
C18-BAC	0.74	0.44	2.06	0.01	0.01	0.02	0.08	0.13	0.47
C8-DADMAC	0.56	0.77	2.88	0.01	0.02	0.06	1.56	2.12	7.92
C10-DADMAC	0.66	0.52	2.24	0.02	0.01	0.03	1.18	2.40	8.38
C12-DADMAC	0.07	0.10	0.50	0.01	0.01	0.03	0.09	0.12	0.45
C14-DADMAC	0.07	0.15	0.52	ND	-	0.01	0.11	0.09	0.38
C16-DADMAC	0.21	0.29	1.07	0.01	0.01	0.02	0.08	0.13	0.46
C18-DADMAC	2.64	1.64	7.57	0.06	0.06	0.23	46.4	47.8	189
C8-ATMAC	ND	-	0.50	ND	-	0.01	0.08	0.13	0.48
C10-ATMAC	ND	-	1.00	0.03	0.01	0.06	1.89	1.87	7.51
C12-ATMAC	0.44	0.18	1.00	0.04	0.05	0.18	0.27	0.16	0.76
C14-ATMAC	0.04	0.08	0.50	0.01	0.01	0.01	0.10	0.14	0.51
C16-ATMAC	3.27	3.74	14.5	0.04	0.02	0.11	0.86	1.29	4.72
C18-ATMAC	0.61	0.78	2.96	0.01	0.01	0.03	0.72	0.62	2.57

The MDL was calculated for each analyte as the sum of the average and three times the standard deviation of blank concentrations for each analyte. In cases, when the analyte was not detected in blanks the limit of quantification was used as the MDL for that analyte.
ND: not detected

Table S3: Mean absolute recoveries (%, with their standard errors) for all QAC analytes in spiked samples.

	Dust				Air				Wristbands			
	5 ng		50 ng		5 ng		50 ng		5 ng		50 ng	
	Mean (n=7)	SE	Mean (n=8)	SE	Mean (n=3)	SE	Mean (n=2)	SE	Mean (n=3)	SE	Mean (n=3)	SE
C6-BAC	116	5	106	5	87	10	73	3	111	3	119	14
C8-BAC	101	2	96	1	99	6	101	2	116	8	117	5
C10-BAC	94	1	93	1	93	5	95	2	101	6	111	8
C12-BAC	105	1	101	2	105	7	116	4	100	11	101	6
C14-BAC	108	5	101	2	102	13	111	4	89	4	94	7
C16-BAC	94	1	98	2	98	2	106	2	112	4	98	5
C18-BAC	101	3	97	4	101	4	86	1	89	15	96	6
C8-DADMAC	85	3	93	3	106	6	116	1	96	6	122	11
C10-DADMAC	99	1	108	5	101	2	106	1	72	16	109	5
C12-DADMAC	100	2	93	4	80	6	72	1	94	3	96	7
C14-DADMAC	104	4	102	3	78	11	80	6	75	7	78	6
C16-DADMAC	102	6	96	4	71	8	73	5	72	16	74	4
C18-DADMAC	98	6	98	4	89	8	83	1	95	9	95	13
C8-ATMAC	104	7	111	8	97	2	96	0	97	10	111	5
C10-ATMAC	111	5	106	4	94	4	97	0	98	30	100	9
C12-ATMAC	99	1	98	2	99	2	104	0	88	8	102	4
C14-ATMAC	101	4	105	3	103	4	101	3	76	7	100	4
C16-ATMAC	99	3	100	3	78	4	83	3	97	13	87	10
C18-ATMAC	109	3	110	5	91	8	89	5	87	9	73	12

Table S4: Spearman coefficients for the correlations among the logarithmically transformed QAC concentrations in dust, air, and wristbands. Correlations were performed for QACs detected in more than 50% of the samples.

		Dust														
		C6-BAC	C8-BAC	C10-BAC	C12-BAC	C14-BAC	C16-BAC	C18-BAC	C8-DADMAC	C10-DADMAC	C12-DADMAC	C14-DADMAC	C16-DADMAC	C18-DADMAC	C8-ATMAC	C10-ATMAC
Wristband	C16-ATMAC	-0.08	0.15	-0.09	-0.11	-0.18	-0.18	-0.16	-0.07	-0.06	-0.07	-0.03	0.06	-0.08	-0.10	-0.14
	C14-ATMAC	-0.12	0.21	-0.17	-0.18	-0.23	-0.22	-0.12	-0.06	-0.06	0.06	-0.07	-0.09	-0.09	-0.12	-0.13
	C12-ATMAC	-0.19	0.21	-0.08	-0.03	-0.09	-0.07	-0.01	0.04	0.07	0.18	0.02	0.06	0.01	0.00	0.03
	C16-DADMAC	-0.14	0.08	-0.14	-0.13	-0.07	-0.04	-0.11	-0.11	0.12	0.19	-0.03	0.09	0.00	-0.04	0.12
	C14-DADMAC	0.22	0.20	0.21	0.38*	0.32	0.20	0.13	0.15	-0.04	0.10	0.07	-0.02	-0.01	0.11	-0.15
	C12-DADMAC	0.11	0.37*	0.24	0.37*	0.35*	0.34*	0.15	0.33*	0.38*	0.58**	0.20	0.25	0.13	0.33*	0.30
	C10-DADMAC	0.02	0.17	0.23	0.31	0.28	0.29	0.07	0.32	0.37*	0.41*	0.17	0.44*	0.27	0.38*	0.42*
	C8-DADMAC	0.28	0.26	0.26	0.36*	0.31	0.26	0.01	0.25	0.13	0.33*	0.32	0.22	0.22	0.30	0.15
	C18-BAC	0.05	0.16	0.06	0.21	0.22	0.12	0.22	-0.06	-0.15	0.12	0.16	-0.03	0.12	-0.06	-0.20
	C16-BAC	0.36*[a]	0.45*	0.38*	0.56**	0.54**	0.46*	0.29	0.34*	0.32	0.51*	0.23	0.11	0.03	0.35*	0.21
	C14-BAC	0.43*	0.48*	0.41*	0.60**	0.58**	0.51*	0.29	0.40*	0.37*	0.53**	0.23	0.11	0.05	0.41*	0.24
	C12-BAC	0.30	0.37*	0.23	0.40*	0.38*	0.34*	0.13	0.20	0.19	0.40*	0.17	0.04	-0.01	0.25	0.06
	C10-BAC	0.36*	0.38*	0.28	0.46*	0.42*	0.34*	0.18	0.20	0.13	0.28	0.20	0.07	0.05	0.26	0.01
	C8-BAC	0.14	0.23	0.03	0.18	0.13	0.07	0.03	0.04	0.03	0.21	0.20	-0.03	-0.07	0.18	-0.03
Air	C8-ATMAC	-0.09	0.06	0.17	0.11	0.04	0.04	0.03	0.11	0.09	-0.10	0.03	0.01	-0.08	0.04	0.13
	C18-DADMAC	0.09	-0.10	0.21	0.12	0.17	0.21	0.13	0.14	-0.05	0.16	0.23	0.19	0.22	-0.03	0.02
	C16-DADMAC	0.24	0.13	0.38*	0.27	0.23	0.27	0.18	0.25	0.10	0.19	0.19	0.31	0.14	0.10	0.12
	C10-DADMAC	0.09	0.28	0.41*	0.30	0.31	0.40*	0.23	0.51*	0.45*	0.40*	0.27	0.21	0.08	0.37*	0.52*
	C18-BAC	0.54**	0.42*	0.47*	0.62**	0.63**	0.56**	0.24	0.41*	0.27	0.37*	0.32	0.05	-0.08	0.43*	0.18
	C16-BAC	0.55**	0.32	0.58**	0.66**	0.67**	0.65**	0.18	0.43*	0.42*	0.38*	0.41*	0.16	0.08	0.53**	0.38*
	C14-BAC	0.52**	0.32	0.61**	0.65**	0.66**	0.66**	0.16	0.47*	0.45*	0.35*	0.43*	0.18	0.12	0.53**	0.42*
	C12-BAC	0.43*	0.32	0.59**	0.50*	0.52**	0.60**	0.16	0.45*	0.46*	0.35*	0.41*	0.11	0.09	0.45*	0.44*
Dust	C18-ATMAC	0.19	0.24	0.38*	0.23	0.28	0.37*	0.31	0.39*	0.31	0.37*	0.40*	0.62**	0.61**	0.32*	0.38*
	C16-ATMAC	0.17	0.35*	0.37*	0.30	0.24	0.31	0.27	0.29	0.16	0.34*	0.35*	0.40*	0.11	0.39*	0.14
	C14-ATMAC	0.18	0.48*	0.53**	0.34*	0.26	0.40*	0.55**	0.47*	0.34*	0.41*	0.39*	0.37*	0.29	0.48*	0.41*
	C12-ATMAC	0.17	0.28	0.24	0.09	0.09	0.20	0.26	0.27	0.18	0.32*	-0.02	0.03	-0.05	0.21	0.15
	C10-ATMAC	0.44*	0.53**	0.67**	0.58**	0.60**	0.72**	0.47*	0.77**	0.91**	0.63**	0.48*	0.38*	0.25	0.86**	1.00
	C8-ATMAC	0.57**	0.61**	0.75**	0.70**	0.69**	0.78**	0.56**	0.81**	0.80**	0.66**	0.65**	0.40*	0.26	1.00	
	C18-DADMAC	0.11	0.05	0.31	0.23	0.24	0.26	0.18	0.19	0.18	0.10	0.45*	0.69**	1.00		
	C16-DADMAC	-0.06	0.07	0.29	0.17	0.19	0.27	0.08	0.32	0.29	0.29	0.59**	1.00			
	C14-DADMAC	0.24	0.36*	0.50*	0.39*	0.39*	0.50*	0.36*	0.51**	0.41*	0.53**	1.00				
	C12-DADMAC	0.36*	0.46*	0.44*	0.42*	0.52**	0.67**	0.37*	0.62**	0.62**	1.00					
	C10-DADMAC	0.49*	0.61**	0.72**	0.67**	0.71**	0.81**	0.47*	0.85**	1.00						
	C8-DADMAC	0.53**	0.69**	0.79**	0.77**	0.78**	0.85**	0.63**	1.00							
	C18-BAC	0.41*	0.63**	0.66**	0.62**	0.56**	0.59**	1.00								
	C16-BAC	0.68**	0.69**	0.88**	0.89**	0.95**	1.00									
	C14-BAC	0.72**	0.63**	0.84**	0.96**	1.00										
	C12-BAC	0.71**	0.67**	0.89**	1.00											
	C10-BAC	0.69**	0.78**	1.00												
	C8-BAC	0.58**	1.00													
	C6-BAC	1.00														

[a] * for $p < 0.05$; ** for $p < 0.001$

		Dust	Air	Wristband

		C12-ATMAC	C14-ATMAC	C16-ATMAC	C18-ATMAC	C12-BAC	C14-BAC	C16-BAC	C18-BAC	C10-DADMAC	C16-DADMAC	C18-DADMAC	C8-ATMAC	C8-BAC	C10-BAC	C12-BAC
Wristband	C16-ATMAC	-0.09	0.15	0.24	-0.21	-0.43*	-0.36*	-0.35*	-0.24	-0.18	-0.04	-0.18	0.04	0.19	0.15	0.13
	C14-ATMAC	0.01	0.16	0.12	-0.18	-0.38*	-0.40*	-0.40*	-0.19	-0.08	-0.10	-0.23	0.08	0.50*	0.35*	0.35*
	C12-ATMAC	0.10	0.31	0.16	-0.09	-0.28	-0.25	-0.26	-0.11	0.03	-0.01	-0.19	0.18	0.60**	0.48*	0.48*
	C16-DADMAC	-0.01	-0.02	0.06	0.20	-0.15	-0.15	-0.06	-0.04	-0.18	-0.09	-0.08	-0.06	0.23	0.18	0.28
	C14-DADMAC	0.23	0.21	0.33*	-0.04	0.12	0.30	0.38*	0.56**	-0.16	0.23	0.01	-0.20	0.48*	0.62**	0.55**
	C12-DADMAC	0.15	0.29	0.42*	0.23	0.03	0.14	0.21	0.28	0.04	-0.07	-0.13	-0.03	0.33*	0.53**	0.57**
	C10-DADMAC	0.12	0.30	0.26	0.36*	0.26	0.29	0.25	0.11	0.44*	0.28	0.00	0.12	0.47*	0.53**	0.57**
	C8-DADMAC	0.00	0.09	0.22	0.28	0.33*	0.42*	0.42*	0.37*	0.12	0.27	0.03	0.01	0.64**	0.64**	0.62**
	C18-BAC	-0.15	0.04	0.09	0.05	0.04	0.16	0.22	0.37*	-0.16	0.18	0.19	-0.03	0.64**	0.59**	0.60**
	C16-BAC	0.12	0.27	0.43*	0.16	0.25	0.40*	0.44*	0.51*	0.03	0.09	-0.02	-0.02	0.59**	0.86**	0.87**
	C14-BAC	0.15	0.29	0.42*	0.16	0.32	0.46*	0.49*	0.57**	0.06	0.23	0.05	-0.03	0.68**	0.92**	0.94**
	C12-BAC	0.10	0.22	0.38*	0.06	0.32	0.44*	0.45*	0.50*	0.02	0.25	0.07	-0.11	0.77**	0.96**	1.00
	C10-BAC	0.07	0.24	0.40*	0.01	0.29	0.45*	0.46*	0.54**	-0.03	0.26	0.06	-0.05	0.79**	1.00	
	C8-BAC	-0.10	0.05	0.16	-0.16	0.06	0.20	0.23	0.39*	-0.08	0.19	-0.15	0.05	1.00		
Air	C8-ATMAC	-0.08	0.22	0.08	-0.17	0.13	0.06	-0.04	-0.11	0.49*	0.17	0.08	1.00			
	C18-DADMAC	0.00	0.22	0.12	0.27	0.40*	0.31	0.25	0.14	0.28	0.61**	1.00				
	C16-DADMAC	0.30	0.38*	0.18	0.22	0.42*	0.36*	0.30	0.24	0.31	1.00					
	C10-DADMAC	0.10	0.35*	0.05	0.19	0.44*	0.28	0.20	-0.08	1.00						
	C18-BAC	0.04	0.00	0.31	0.09	0.49*	0.74**	0.83**	1.00							
	C16-BAC	-0.01	0.05	0.29	0.14	0.82**	0.96**	1.00								
	C14-BAC	0.00	0.12	0.26	0.09	0.91**	1.00									
	C12-BAC	0.10	0.20	0.17	0.14	1.00										
Dust	C18-ATMAC	0.30	0.37*	0.29	1.00											
	C16-ATMAC	0.36*	0.63**	1.00												
	C14-ATMAC	0.65**	1.00													
	C12-ATMAC	1.00														

[a] * for $p < 0.05$; ** for $p < 0.001$

		Wristband										
		C14-BAC	C16-BAC	C18-BAC	C8-DADMAC	C10-DADMAC	C12-DADMAC	C14-DADMAC	C16-DADMAC	C12-ATMAC	C12-ATMAC	C16-ATMAC
Wristband	C16-ATMAC	0.07	0.13	0.01	0.03	-0.02	0.31	0.00	0.31	0.59**	0.74**	1.00
	C14-ATMAC	0.29	0.30	0.24	0.21	0.13	0.38*	0.16	0.32	0.87**	1.00	
	C12-ATMAC	0.42*	0.42*	0.35*	0.35*	0.38*	0.54**	0.23	0.42*	1.00		
	C16-DADMAC	0.26	0.32	0.24	0.06	0.16	0.46*	0.00	1.00			
	C14-DADMAC	0.57**	0.50*	0.50*	0.28	0.21	0.33*	1.00				
	C12-DADMAC	0.69**	0.75**	0.29	0.44*	0.48*	1.00					
	C10-DADMAC	0.59**	0.53**	0.26	0.68**	1.00						
	C8-DADMAC	0.60**	0.54**	0.42*	1.00							
	C18-BAC	0.56**	0.55**	1.00								
	C16-BAC	0.96**	1.00									
	C14-BAC	1.00										

[a] * for $p < 0.05$; ** for $p < 0.001$

Table S5: Median concentrations (ng/g/day) and the results of the comparative analysis for the QACs detected in more than half of the resident and staff wristbands.

	Median Residents	Median Staff	Rel. Conc.[a]	*p-value*
C8-BAC	0.060	0.256	4.23	0.013
C10-BAC	0.177	0.751	4.24	0.011
C12-BAC	47.7	129	2.70	0.020
C14-BAC	90.3	215	2.38	0.007
C16-BAC	21.4	53.8	2.51	0.002
C18-BAC	0.169	0.285	1.69	N.S.
C8-DADMAC	9.48	7.67	0.81	N.S.
C10-DADMAC	9.01	14.7	1.63	N.S.
C12-DADMAC	1.77	4.35	2.45	0.006
C14-DADMAC	0.232	0.103	0.440	N.S.
C16-DADMAC	0.806	0.538	0.670	N.S.
C12-ATMAC	0.338	0.268	0.790	N.S.
C14-ATMAC	0.120	0.146	1.22	N.S.
C16-ATMAC	2.86	2.010	0.700	N.S.

[a] Rel. Conc. represents the ratio of median QAC concentrations in staff- to resident-worn wristbands. 'N.S.: not significant at $p < 0.05$.

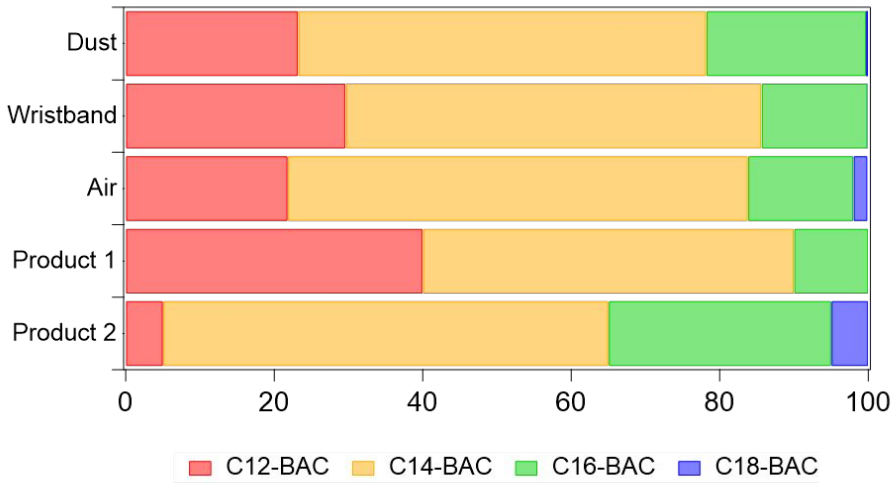

Figure S1: Comparison of percent contribution of C12-, C14-, C16-, and C18-BAC to the total QAC concentrations in dust, wristbands, air, and in products registered by the US EPA as antimicrobial products as of March 14, 2017 (CAS: 53316-76-0 [Product 1] and CAS: 68424-85-1 [Product 2]).[1]

References

(1) United States Environmental Protection Agency. Alkyl dimethyl benzyl ammonium chloride (ADBAC) final work plan. **2017**.
